# *Culex pipiens* Development Is Greatly Influenced by Native Bacteria and Exogenous Yeast

**DOI:** 10.1371/journal.pone.0153133

**Published:** 2016-04-07

**Authors:** Leonardo M. Díaz-Nieto, Cecilia D´Alessio, M. Alejandra Perotti, Corina M. Berón

**Affiliations:** 1 Instituto de Investigaciones en Biodiversidad y Biotecnología (INBIOTEC)–CONICET, Mar del Plata–Argentina and Fundación para Investigaciones Biológicas Aplicadas (FIBA), Mar del Plata, Argentina; 2 Fundación Instituto Leloir- Instituto de Investigaciones Bioquímicas de Buenos Aires (IIBBA)–CONICET, Argentina and Facultad de Ciencias Exactas y Naturales, UBA, Buenos Aires, Argentina; 3 Ecology and Evolutionary Biology, School of Biological Sciences, University of Reading, Reading, United Kingdom; Swedish University of Agricultural Sciences, SWEDEN

## Abstract

*Culex pipiens* is the most cosmopolitan mosquito of the Pipiens Assemblage. By studying the nature of interactions between this species and microorganisms common to its breeding environment we can unravel important pitfalls encountered during development. We tested the survival rate of larval stages, pupae and adults of a *Cx*. *pipiens* colony exposed to a variety of microorganisms in laboratory conditions and assessed the transmission to offspring (F1) by those organisms that secured development up to adulthood. Three complementary experiments were designed to: 1) explore the nutritional value of yeasts and other microorganisms during *Cx*. *pipiens* development; 2) elucidate the transstadial transmission of yeast to the host offspring; and 3) to examine the relevance of all these microorganisms in female choice for oviposition-substratum. The yeast *Saccharomyces cerevisiae* proved to be the most nutritional diet, but despite showing the highest survival rates, vertical transmission to F1 was never confirmed. In addition, during the oviposition trials, none of the gravid females was attracted to the yeast substratum. Notably, the two native bacterial strains, *Klebsiella* sp. and *Aeromonas* sp., were the preferred oviposition media, the same two bacteria that managed to feed neonates until molting into 2^nd^ instar larvae. Our results not only suggest that *Klebsiella* sp. or *Aeromonas* sp. serve as attractants for oviposition habitat selection, but also nurture the most fragile instar, L1, to assure molting into a more resilient stage, L2, while yeast proves to be the most supportive diet for completing development. These experiments unearthed survival traits that might be considered in the future development of strategies of *Cx*. *pipiens* control. These studies can be extended to other members of the Pipiens Assemblage.

## Introduction

In the development of new strategies for the control of disease-carrying mosquitoes, the study of food sources, particularly microorganisms that might be critical for securing the survival of developmental stages of these insects, is essential. Mosquitoes exhibit complete metamorphosis with development accomplished in aquatic habitats where the eggs are laid, hatched, and passed through four larval instars and a pupal stage until molting into imagoes. In many species, to secure and complete oogenesis, females have to take a blood-meal after copulation; while larvae are filter feeders taking their food from the water bodies they inhabit [1]. According to Laird [2] the larval food of mosquitoes consists of a range of non-living and living resources as organic detritus, bacteria, unicellular algae, different protozoa, micro-metazoa and small filamentous algae. In addition, mosquito larvae seem not to discriminate the type of food they eat [3].

The nutritional function of yeasts has been demonstrated in some insects. Yeasts provide enzymes which help the host to digest essential amino acids, vitamins and sterols. It has also been reported that yeasts play some role in detoxification of toxic metabolites originated in the host’s diet, however, this mainly relates to insects that feed on plants or their fluids [4]. In some insects, yeasts have been identified in the gut as well as inside special organs known as mycetocytes. In relation to vertical transmission to the progeny only a few species of yeasts had been confirmed for insects in general, by being smeared on to the egg shell for future consumption by the 1^st^ instar or hatching larva [5–7]. Only a few species of yeast have been described from mosquitoes, and their function and the possibility of being transmitted to the next generation is not clear [6,8–10]. *Candida intermedia*, *Hanseniaspora uvarum* and *Wickerhamomyces anomalus* (= *Pichia anomala*) were found in newly emerged lab-reared *Anopheles stephensi* [11]. *W*. *anomalus* was found to live in association with some populations of *An*. *stephensi*, *Anopheles gambiae*, *Aedes* (*Stegomyia*) *aegypti and Aedes* (*Stegomyia*) *albopictus*, and further localization studies in the midgut and reproductive systems might suggest multiple transmission patterns of the yeast [10]. *Pichia* and *Candida* species were isolated from gut diverticulum and identified from ovaries of *Ae*. *aegypti* but no further investigation addressed the role nor their transmission patterns [9,12]. In an in-depth characterization of mosquito-associated yeasts, including hosts such as *Cx*. *pipiens*, *Anopheles maculipennis* and four *Aedes* (*Ae*. *cataphylla*, *Ae*. *communis*, *Ae*. *cantans*, *Ae*. *cinereus*), Ignatova *et al*. [8] identified 18 species out of 7 genera of yeasts; however, none of the 18 yeast strains were pathogenic to any bloodsucking mosquito species studied; and experiments of larvae infected with yeasts grew and developed significantly faster as compared to control, encompassing reduction of the developmental time for all mosquitoes reared on yeast flora [8]. Therefore, they concluded that yeasts serve as a highly nutritional food item for mosquito larvae, being a regular component of the mosquito microbiota. Despite their diversity and prevalence, yeast role in mosquito’s life cycle is still poorly understood. In addition, for the past two decades research has been mainly focused on vectors of malaria and dengue, such as *Anopheles* and *Aedes* species, while studies on other more widespread vectors, like those in the Pipiens Assemblage remain neglected.

On the other hand, mosquito-bacteria associations have been the aim of several studies from attempts to understand symbiotic interactions, exploring their application in paratransgenic methods up to the discovery of new entomopathogens. In the last two decades the characterization of the microbiota of mosquitoes has been the focus of relevant discoveries, especially microorganisms localized in the gut and salivary glands, particularly some bacteria inhabiting the midgut along with blood digestion [13]. It has been shown that bacterial communities may differ according to different breeding habitats of mosquitoes in their larval stages [14,15], according to the developmental stage of the insect [16], or also according to the species of mosquito analyzed [17]. Some of them, like *Enterobacter* sp., have been suggested to be transovarially transmitted to the host offspring [12]; and *Klebsiella* sp. and *Aeromonas* sp. were proposed as gut symbionts and not just as a food source [18–21]; particularly *Aeromonas* sp. is an important microbiota component of other *Culex* species like *Culex tarsalis* [14,15].

*Cx*. *pipiens* is the main species characterizing the Pipiens Assemblage, the most widespread group of mosquitoes in the world [22]. The group comprises the principal vectors of St. Louis encephalitis virus (SLEV), West Nile virus (WNV), Rift Valley Fever virus (RVFV), filarial worms and some other wildlife pathogens like bird malaria [23]. In Argentina the Pipiens Assemblage includes *Cx*. *pipiens* and *Culex quinquefasciatus*, these species are found in all biogeographic regions of the country [24,25], being both competent vectors of WNV and SLEV [26,27].

With the aim of analyzing microbiota-mosquito interactions within the Pipiens Assemblage we have explored the nutritional value of yeasts and other commonly associated microorganisms during *Cx*. *pipiens* development as well as addressed transmission to offspring. The relevance of these microorganisms in female choice for oviposition-substratum was also investigated, as females might want to secure food sources, making them immediately available for the most vulnerable stage, the 1^st^ instar larvae.

## Materials and Methods

### Ethics Statement

The protocol for blood feeding mosquitoes on mice was reviewed and approved by the Animal Experimental Committee at the Faculty of Exact and Natural Sciences, Mar del Plata University (Institutional Committee on Care and Use of Experimental Animals (CICUAL) N° 2555-04-14). The mice were handled in strict accordance with National Health Service and Food Quality (SENASA) guidelines Argentina and with the 2011 revised form of The Guide for the Care and Use of Laboratory Animals published by the U.S. National Institutes of Health.

### Laboratory-reared mosquitoes

Analyzed mosquitoes were obtained from a one-year-old colony of *Cx*. *pipiens* reared in the insectary of the Biological Control Laboratory of the INBIOTEC-CONICET, FIBA (Argentina). They were maintained at standard conditions of 24°C and 80 ± 5% relative humidity (RH), photoperiod (12 h light: 12 h dark) and in near-axenic settings during both the developmental and adult stages. For near-axenic conditions larvae, pupae and eggs were externally sterilized as described below and the females were exposed to sterile distilled water inside plastic containers for the oviposition. After eggs hatching, larvae were grown in different plastic containers, filled with sterile distilled water and fed with the different microorganisms offered for each treatment, and with commercial fish food (Shulet® Carassius) as a control diet. Adult stages were provided with sterile 10% sucrose solution and the females with fresh blood from mice.

### Microorganism strains, media composition and growth conditions

*S*. *cerevisiae* (*MATa*, *his3Δ1*, *leu2Δ0*, *met15Δ0*, *ura3Δ0*, *PIL1*::*GFP*), native *Klebsiella* sp. and *Aeromonas* sp. isolated from a *Cx*. *pipiens* laboratory colony were grown at 28°C in yeast peptone dextrose medium supplemented with adenine, YPDA media plates [28] or potato dextrose agar, APG medium (Britania). The cyanobacteria *Anabaena* PCC 7120 and *Synechocystis* PCC 6803, and the microalgae *Chlorella sorokiniana* RP were grown at 28°C in BG11 medium [29].

*S*. *cerevisiae* GFP-labelled strain was a generous gift from Dr. Alejandro Colman Lerner (School of Sciences, University of Buenos Aires); *Anabaena* 7120 and *Synechocystis* 6803 were provided by Institute Pasteur (Paris) and *C*. *sorokiniana* strain RP is part of the FIBA culture collection. Mosquito native bacteria, *Klebsiella* sp. and *Aeromonas* sp. were isolated during the development of this research using classical microbiological methods, by cultivating in YPDA medium, with samples obtained from homogenates of larvae, pupae and adults of *Cx*. *pipiens*. Each microorganism was grown in their specific media harvested by centrifugation, washed twice with 10% TE buffer (100 mM Tris HCl pH 7, 10 mM EDTA), lyophilized and later used as larval food.

### Evaluation of the nutritional value of microorganisms on the development of *Cx*. *pipiens*

In order to analyze the function of yeasts in the development of *Cx*. *pipiens* mosquitoes, different microorganisms were provided as food source from neonate to L4 larvae to assess growth and development of the different developmental stages. For that, egg rafts from *Cx*. *pipiens* laboratory breeding were washed with 0.01% of non-ionic detergent (Biopack) and then with sterile distilled water for three times to remove any contaminants by external microorganisms. After this, the eggs were placed in plastic containers with sterile distilled water until the hatching of neonate larvae. Two diet assays were carried out, experiment 1: groups of 100 larvae divided into subgroups of 20 were transferred to plastic containers with 100 mL of sterile distilled water in each of them and were fed with different microorganisms: yeasts (GFP-labelled *S*. *cerevisiae*), microalgae (*C*. *sorokiniana*), cyanobacteria (*Anabaena* 7120 and *Synechocystis* 6803), bacteria (*Klebsiella* sp. and *Aeromonas* sp.), and fish food as a control diet (Shulet® Carassius, composed of fish, meat, liver and soybeans flours, sea algae, cereals, wheat germ, casein, baking powder, egg powder, dehydrated spinach, fish oil with butylhydroxytoluene). The fish food used as control food for the experiments has yeast as part of its composition; however, we verified that yeast was unviable by culturing it in YPDA medium. One milligram of each lyophilized cell culture was used to feed each group of larvae. Developmental stage and number of surviving individuals per treatment were monitored every 24 h. Experiment 2: To have consistency in the analysis of the results, the experiment was repeated three more times; but in this step, larvae from the same egg raft were used for each repetition, each raft has an average of 150 eggs. Thereby, 20 larvae were transferred to plastic containers with 100 mL of sterile distilled water in each of them and were fed with the same food items as in experiment 1.

### Transstadial transmission of yeast in *Cx*. *pipiens*

In our studies, no native yeasts were detected in the *Cx*. *pipiens* laboratory colony population, due to the lack of success isolating any native yeast from our *Cx*. *pipiens* laboratory colony, we decided to pursue the nutrition and transmission experiments by applying the same organism, *S*. *cerevisiae*, a model yeast used in many bioassays and analyze if it could be transmitted to the insect progeny. The *S*. *cerevisiae* strain used had the advantage of being available labelled with GFP, therefore, transstadial transmission could be followed *in situ* (host) throughout development by just incorporating it as a main food item from neonate larvae onwards.

To follow the presence and location of the labelled yeast in the different mosquito stages along the life cycle including the offspring eggs, 800 *Cx*. *pipiens* larvae from the laboratory bred mosquitoes were fed with GFP-labelled *S*. *cerevisiae* during the whole larval development using a 24 h yeast culture. Insects of each individual stage were observed by fluorescence microscopy and GFP-labelled cells were detected in the digestive tracts in the four larval stages by fluorescent microscopy, in concordance with the expected time of fluorescent emission by the yeast lasting up to 48–72 h after being cultured in rich media (YPDA). To determine if the yeast stays alive during pupal and adult stages, and F1 eggs, all the developmental stages of the insects were homogeneized in saline solution and grown in YPDA medium plates, incubated at 28°C for 24–48 h and used for molecular analysis. Before each treatment, mosquitoes were rinsed and sterilized superficially in the following solutions: sodium hypochlorite (5% for 2 min) and rinsed twice with sterile distilled water (2 min). Internal symbionts, such as endosymbionts or any vertically transmitted bacteria cannot be removed by these methods. Additionally, aliquots of 100 μL from the last wash (sterile distilled water) were grown in YPDA and APG media as a control of the efficiency of the surface sterilization process.

### Light and fluorescence microscopy

GFP-labelled *S*. *cerevisiae* colonies and cells were examined by light and fluorescence microscopy using a Nikon E600 microscope with 10X and 100X objectives respectively. Images were captured by digital camera Olympus DP72, using Cellsens Entry imaging software.

### Yeast detection and treatment for DNA extraction from F1 eggs

In order to determine the yeast growth from the F1 eggs inoculated by females during oviposition and to detect yeast transferred by the female into the eggs, 100 L1 larvae, placed in 100 mL of deionized water, were fed every other day with 1 mL of GFP-labelled *S*. *cerevisiae* strain grown in YPDA media until OD_600_ = 1 (about 3 x 10^7^ cells/mL). The larvae were washed when they reached the pupal stage, transferred into sterile water and maintained in separate cages until adult emergence. Newly emerged mosquitoes were fed on a sterile cotton pad soaked with sterilized 10% sucrose solution (renewed every 24 h) and females were supplied with blood after their sexual maturity (5 days or after seeing mating). Two days after blood meal, they were transferred into a rearing cage and a plastic container with sterile water for egg laying. Each oviposition was immediately collected and grown in YPDA culture medium for the isolation of labelled yeast or DNA extraction for yeast detection into the eggs by the GFP amplification.

### DNA extraction and PCR-based analysis

DNA extraction from washed insects of each developmental stage and F1 eggs were conducted under sterile conditions and performed with the PureLink™ Genomic DNA Mini Kit (Invitrogen) and DNeasy Blood and Tissue Kit (Qiagen). Genomic DNA extractions from yeast and bacteria cultures were done by conventional techniques [28]. The PCR reactions were carried out by using 0.2 μg of DNA template in a reaction mixture (total volume, 25 μL) containing each deoxynucleoside triphosphate at a concentration of 400 μM, each primer at a concentration of 0.5 μM, and 0.5 U of Taq DNA polymerase dissolved in the corresponding reaction buffer (Promega). Amplifications were performed with a Mastercycler® Epgradient (Eppendorf) under the following conditions: 10 min of denaturation at 94°C, followed by 30 cycles of denaturation for 30 s at 94°C, annealing for 45 s at 50–55°C depending on the primers used, extension for 45 s at 72°C and an extra extension step of 1 min at 72°C. Primer pairs, yeast-F1 and yeast-R1 [11] and NS1 and FS2 [30], were used to amplify mosquito associated yeasts. Small subunit rRNA genes (16S rDNA) from the native bacteria were amplified by PCR using universal bacteria specific primers, 27f and 1495r [31]. For the amplification of GFP gene fragment, which length is about 700 bp, the primer pair GFPFw (5’ GGAGAAGAACTTTTCACTGGAG 3’) and GFPRev (5’ CATCCATGCCATGTGTAATCCTAG 3’) was used under the following amplification conditions: 90 s of denaturation at 94°C, followed by 40 cycles of denaturation for 15 s at 94°C, annealing for 60 s at 50°C, and extension for 90 s at 72°C and an extra extension step of 1 min at 72°C. PCR products were run on 1% agarose gel-electrophoresis, analyzed using a gel imaging system (Photodyne Incorporated, Hartland, WI, USA) and were sequenced at Macrogen (Korea). BLASTn (http://blast.ncbi.nlm.nih.gov/Blast.cgi) was used for DNA sequence analysis.

### Impact of microorganisms on oviposition substratum preferences by *Cx*. *pipiens* female

We carried out oviposition tests offering different substrates inoculated with specific microorganisms to females for laying eggs. Adult mosquitoes were kept in standard 30x30x30 cm rearing cages at INBIOTEC insectary at 25–27°C; 65–70% RH and photoperiod of 12 h light: 12 h dark, and fed with a 10% sucrose solution *ad libitum*. Five-day-old males and females were placed together since their emergence and then, after copulation, females were fed with fresh blood during 30 min per day. Blood-fed females were collected with a manual aspirator, kept in standard 20x20x20 cm rearing cages and provided with 10% sucrose solution. After two days, Petri dishes of 5 cm of diameter filled with 5 mg of different lyophilized microorganisms (*S*. *cerevisiae*, *C*. *sorokiniana*, *Anabaena* 7120, *Synechocystis* 6803, *Klebsiella* sp. and *Aeromonas* sp.) resuspended in 10 mL of sterile water were put into the cages. A Petri dish with 10 mL of sterile water was used as control assay; plates with the different oviposition substrates were placed randomly in rearing cages and so the cages were placed randomly on racks in a room with the same environmental conditions as in the insectary. Laying of eggs on the surface of the different treatments and controls were counted after 24 h exposure period.

### Statistical analysis

**Fig 1:**
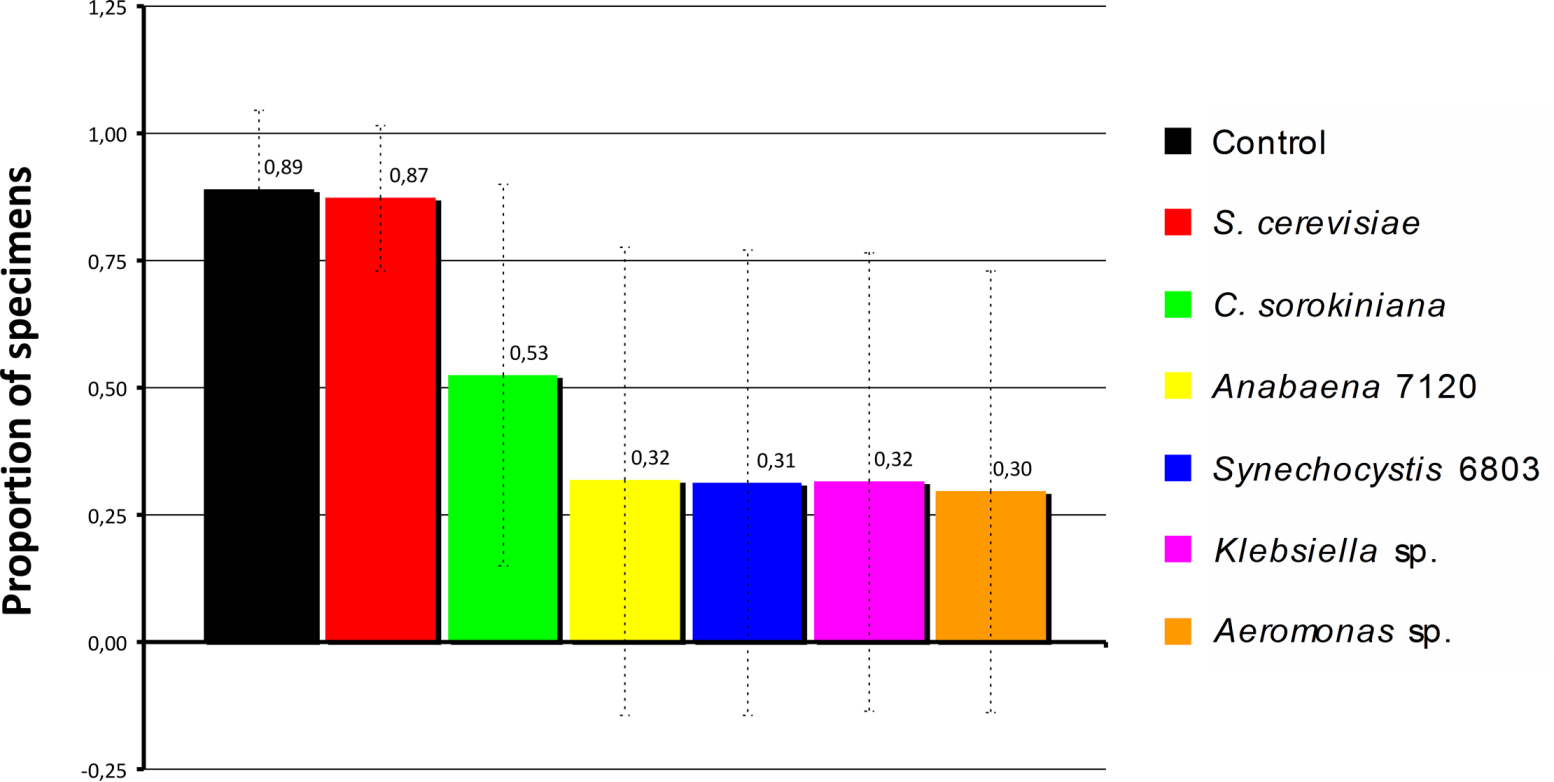
Survival rates. Bars representing mean of survival rate by treatment (microorganism), lines indicate standard deviation (SD) and show great dispersal for bars 3 to 7. N_samples included in the assay_ = 24, N_individuals at the experiment started_ = 160. Treatments: *S*. *cerevisiae*, *C*. *sorokiniana*, *Anabaena* 7120, *Synechocystis* 6803, *Klebsiella* sp. and *Aeromonas* sp.

Final rates of survival were calculated as the fraction of the total resulting specimens (survivors) over the total of specimens at the start of the two nutritional experiments. Although survival data were not normally distributed, and this can be visualized in Fig 1, bars or treatments 3 to 7, we still compared the analysis of the non-parametric tests (used in the survival curves, below) with parametric tests, assuming dependency of the data (from the same original lab population). This was done at particular time steps. Therefore, average survival rates for given days/stages were compared by Student’s paired *t* Test for *S*. *cerevisiae* vs control-food only (and only day 25) and by ANOVA (with Bonferroni correction for multiple comparisons) for all treatments (only days 10 and 25). Survival functions were generated by the non-parametric Kaplan-Meier survival estimator [32]. Preferences for oviposition substratum were analyzed with Cochran's Q Test [33].

## Results

### Evaluation of the nutritional value of microorganisms on the development of *Cx*. *pipiens*

Larvae fed with *S*. *cerevisiae* showed the highest survival rate of all treatments. No significant difference was observed between the mean survival rates when fed with *S*. *cerevisiae* and fish food (*P* = 0.315) (Fig 1).

**Fig 2:**
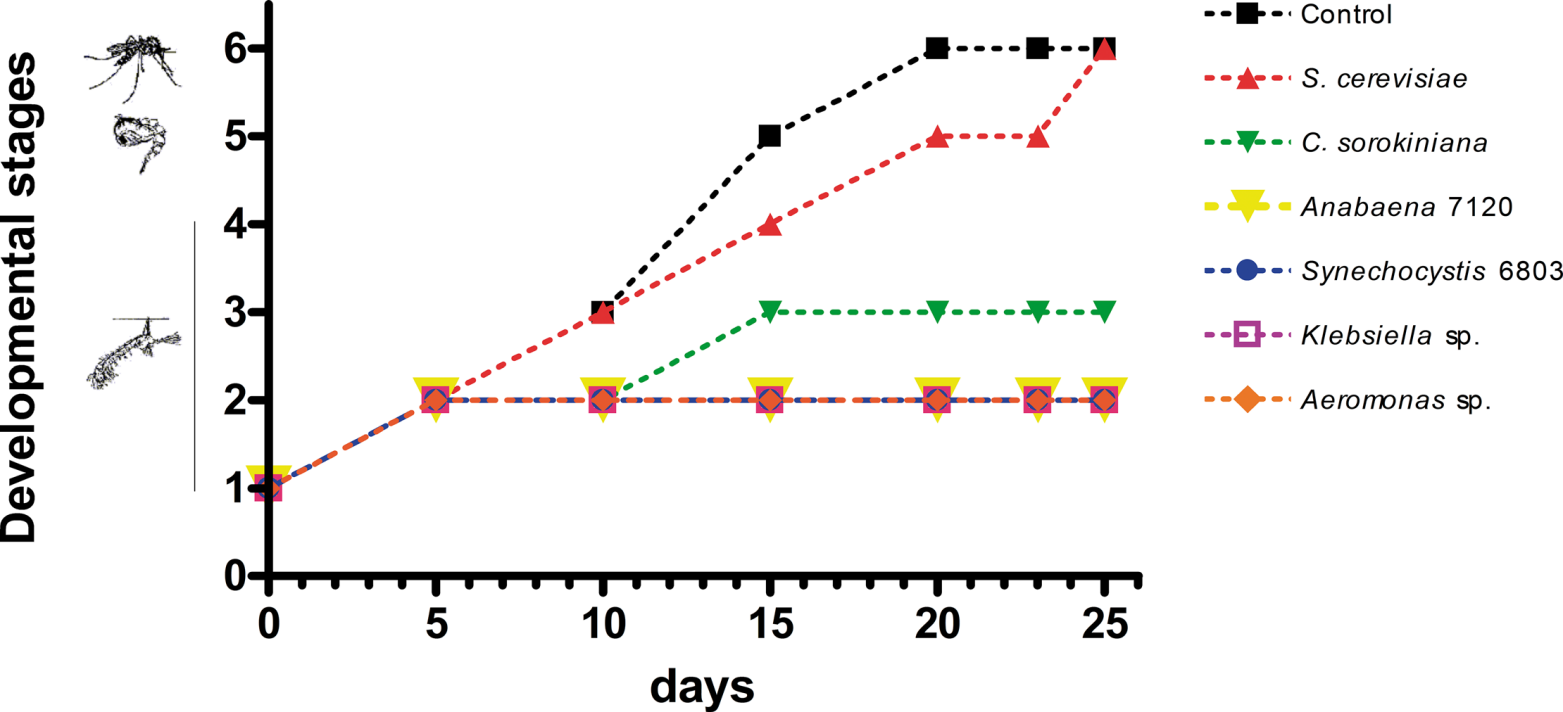
Developmental time of the immature stages of *Cx*. *pipiens* fed with different microorganisms. 100 larvae of *Cx*. *pipiens* were placed in groups of 20 and fed on different microorganisms (*S*. *cerevisiae*, *C*. *sorokiniana*, *Anabaena* 7120, *Synechocystis* 6803, *Klebsiella* sp. and *Aeromonas* sp.). The larval development was observed at 5, 10, 15, 20, 23 and 25 days after feeding with each microorganism. The developmental status was determined based on the developmental state of more than 50% of individuals of each treatment.

Fig 1 shows standard deviations for each treatment in all sampling days (survival). Large variances were particularly observed for *C*. *sorokiniana*, *Anabaena* 7120, *Synechocystis* 6803, *Klebsiella* sp. and *Aeromonas* sp. (bars 3 to 7). These datasets showing the highest deviations, due to high mortality were not compared; therefore, the parametric statistical analysis (considering data dependency) were only performed on days 10 and 25 (survival) (S1 Table). Specimens exposed to cyanobacteria, microalgae and native bacteria failed to develop into adult stage, showing very low survival rates, while those on yeast and fish food succeeded (Fig 2 and S1 Fig).

**Fig 3:**
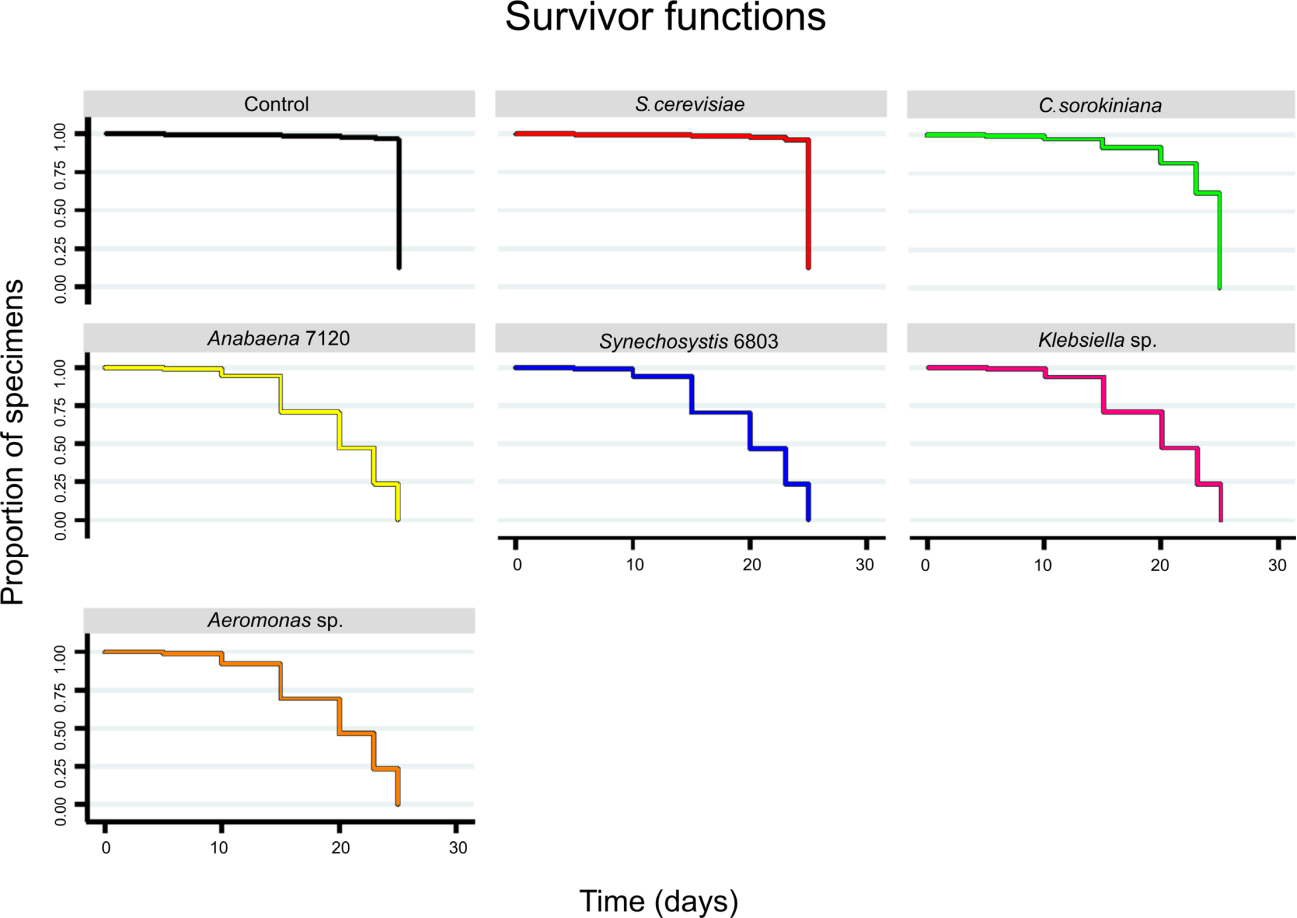
Survival curves. Kaplan-Meier survival curves per treatment (Treatment = microorganism, N_individuals at the experiment started_ = 160, α = 0.05) were estimated from total individuals of each treatment (*S*. *cerevisiae*, *C*. *sorokiniana*, *Anabaena* 7120, *Synechocystis* 6803, *Klebsiella* sp. and *Aeromonas* sp.).

The shortest development times were achieved when larvae were fed with yeast and control-food (Fig 2 and S1 Fig): adulthood was reached after 20 and 25 days when they were fed with control-food and yeast, respectively, showing in both cases a similar survival rate (Fig 3).

*Cx*. *pipiens* fed on microalgae were only able to survive until larval stage 3 (L3), after 25 days of treatment, showing also a rapid decrease in survival rate after 10 days. When fed with cyanobacteria and with the two native bacterial strains the rate of larval survival drastically dropped (Figs 2 and 3 and S1 Fig), reaching at last second larval instar and dying on day 15^th^. On the other hand, *in vivo* observations demonstrate that larvae were able to ingest microalgae and cyanobacteria but after a few minutes these microorganisms were excreted without being digested (S1 Video).

### Transstadial transmission of yeast in *Cx*. *pipiens*

*In situ* microscopic examination of specimens allowed the detection of the labelled *S*. *cerevisiae* in all larval stages with clear visualization in midgut and in gastric ceca (S2 Fig). Signal of GFP-labelled yeast was detected up to 72 h in post-feeding larvae (L1, L2, L3, L4); none of the other stages—pupae, imagoes (females-males) including F1 eggs and neonates—showed fluorescent signal (Table 1).

**Table 1:** *S*. *cerevisiae* GFP-label detection in different developmental stages of *Cx*. *pipiens* by the identification of the presence of fluorescent signal observed under fluorescence microscopy, grown in YPDA culture medium and the amplification of the GFP gene by PCR.

	Fluorescence microscopy	Yeast GFP-label grown in YPDA medium	GFP gene amplification
Developmental stage	Yeast detection	Yeast detection	GFP gene detection
**Larvae 1**	+(20/20)	+(20/20)	+(17/20)
**Larvae 2**	+(20/20)	+(20/20)	+(19/20)
**Larvae 3**	+(20/20)	+(20/20)	+(15/20)
**Larvae 4**	+(20/20)	+(20/20)	+(17/20)
**Pupae**	-(0/20)	+(14/20)	+(13/20)
**Adult female**	-(0/20)	-(0/20)	+(8/20)
**Adult male**	-(0/20)	-(0/20)	-(0/20)
**Eggs F1**	-(0/10)	-(0/10)	-(0/10)
**Larvae 1 F1**	-(0/20)	-(0/20)	-(0/20)

(+): Positive detection of *S*. *cerevisiae* GFP-label. (-): Negative detection of *S*. *cerevisiae* GFP-label. In brackets: (positive detection of *S*. *cerevisiae* GFP-label / total analyzed).

On the other hand, PCR screenings indicated the presence of the GFP gene up to adult females; no adult males nor eggs and larvae 1 F1 carried GFP-labelled *S*. *cerevisiae* (Table 1 and S3 Fig). Finally, after growing each specimen’s homogenate in YPDA culture medium, we only observed yeast grown from larvae and pupae with negative results from adults, F1 eggs and larvae L1 F1 (Table 1). GFP-labelled *S*. *cerevisiae* was not detected in F1 eggs by any methodology used (Table 1).

### Native bacteria detection

We only isolated bacterial cultures when homogenates from larvae, pupae and F1 eggs obtained from larval stages selectively fed on GFP-labelled *S*. *cerevisiae* or on fish-food were grown on YPDA medium. These bacterial colonies (mosquito native bacteria) were isolated (S4 Fig) and molecularly identified as *Klebsiella* sp. and *Aeromonas* sp. by 16S rRNA PCR amplification.

### Impact of microorganisms on oviposition substratum preferences by *Cx*. *pipiens* female

**Fig 4:**
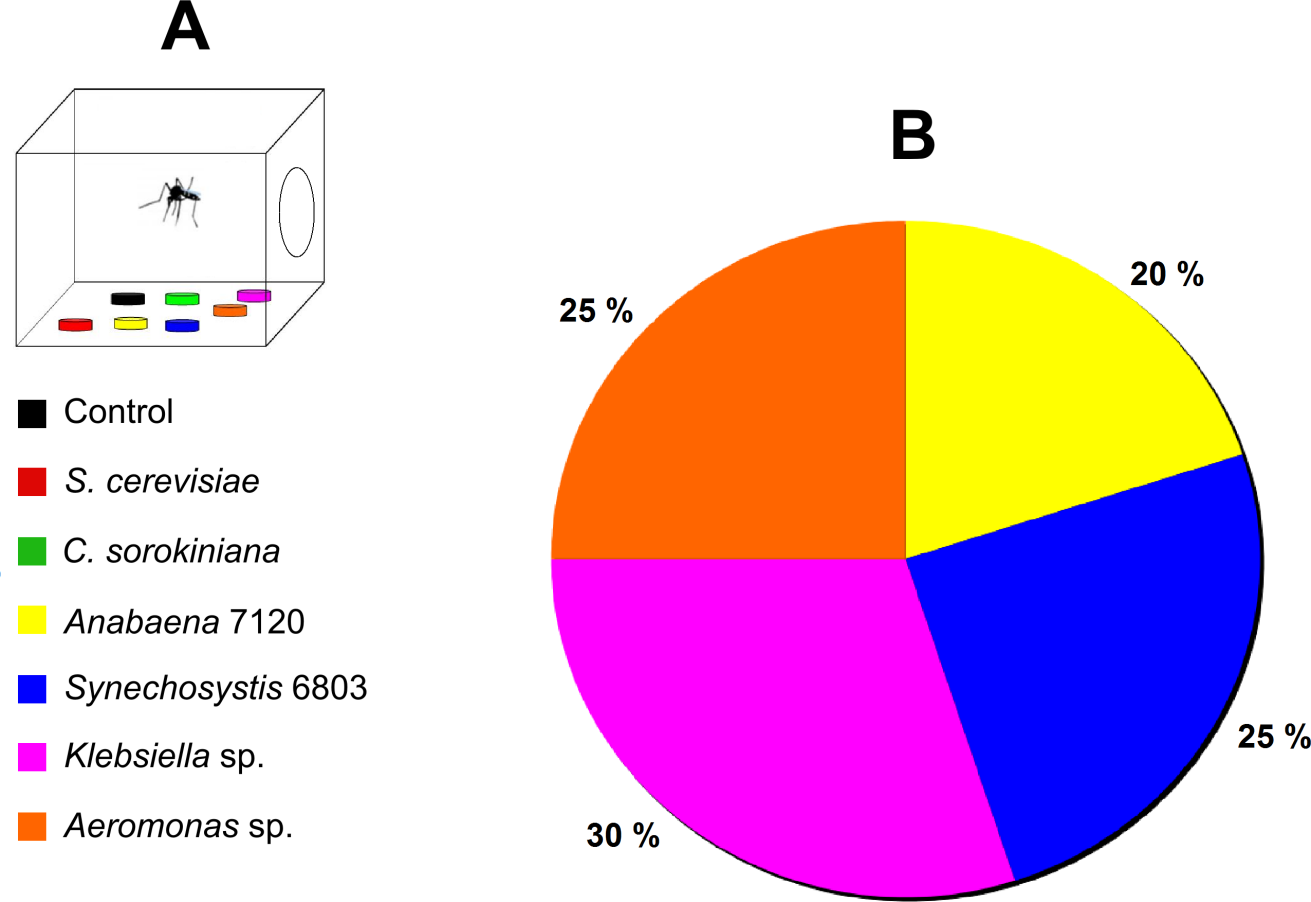
Oviposition substratum choice. (A) Diagram of oviposition preference assays with female mosquitoes. (B) Percentage (%) of oviposition substratum (microorganism) selected by gravid females. N_eggs raft_ = 20.

Mosquito females greatly ignored yeasts as oviposition substratum; instead, the most attractive substratum was *Klebsiella* sp. (*X*^2^_6_ = 15.996, *P* = 0.014), closely followed by *Aeromonas* sp. and *Synechosystis* 6803 (Fig 4). In addition, females chose the two cyanobacteria substrata.

## Discussion

In this work we demonstrate that *Cx*. *pipiens* larvae are able to complete development up to adulthood when they were fed with a yeast treatment and with fish food used as control food, showing that yeast is as nutritional as the control or regular mosquito rearing-food. These results were expected since brewer yeast is a common compound included in the fish food brands used in this work. Brewer yeast, *S*. *cerevisiae* is also systematically used for laboratory mosquito rearing trials [34,35].

According to Chapman [36] variations in the quantity or quality of an acceptable diet can have radical effects on insects’ development and lifespan. Mosquitoes feed on a diverse range of food items. Gut analysis of food ingested by mosquito larvae allowed the detection of cyanobacteria [14,15,19], organic detritus, algae, protozoans and other microorganisms [3,37]. Our results support the mixed diet feeding, taking into account that we compared *S*. *cerevisiae* as food source of *Cx*. *pipiens* with other microorganisms such as algae, model cyanobacteria strains and native bacteria. Howland [38] suggested that algae ingested by many species of mosquito larvae could be an important part of their diet, but generally would not be digested and Marten [39] supports that certain species of green algae from the order Chlorococcales kill mosquito larvae primarily because they are indigestible. In concordance with these researchers, we observed feeding larvae behavior *in vivo*, on microalgae and cyanobacteria, and determined that, although these microorganisms were ingested, they were excreted (un-digested) after a few minutes (S1 Video); this behavior was not observed in larvae fed with bacteria, yeast or control-food. Larvae fed with microalgae, cyanobacteria or native bacterium did not complete their development. Clements [3] already proposed that mosquito larvae are not capable of discriminating food items. Lack of food selection strategies suggests no adaptation to intake or filtering, as a result, larvae are generalist filters.

Studies of the interaction between some yeasts and larval and adult stages of *Drosophila* sp. indicated that yeasts are necessary for optimal development due to their nutritional role, providing enzymes for digestion [7]. Ignatova *et al*. [8] were the first realizing that yeast alone can nurture mosquito larvae. Our results confirm these early findings: yeast was the only food item (other than control-food) to successfully nurture developmental instars until the adult stage and to show at the same time the highest survival rate. However, with respect to the vertical transmission of *S*. *cerevisiae*, we could not demonstrate the transmission to the *Cx*. *pipiens* progeny, despite being essential food for development. Fluorescent signal of GFP-labelled yeast was detected until L4 possibly because the signal vanishes around 72 h, so the GFP gene was detected by PCR, but gene detection does not imply that the yeast was alive, for that reason, yeast from each of the mosquito stages was isolated in YPDA medium, obtaining the growth of colonies until pupa stage, but not from adults, F1 eggs nor from L1 larvae.

The two bacteria detected in the mosquitoes used in this work, *Klebsiella* sp. and *Aeromonas* sp. were previously isolated from different mosquito species, including some in the Pipiens Assemblage. Despite conducting all experiments under sterile conditions (external sterilization of specimens), native bacteria *Klebsiella* sp. and *Aeromonas* sp. could be transferred onto the next stages including the F1, confirming the vertical transmission of these microorganisms in concordance with the results of Coon, from *Ae*. *aegypti* [16]. Demaio *et al*. [40] observed in *Aedes triseriatus* (now renamed *Ochlerotatus* (*Protomacleaya*) *triseriatus*), *Cx*. *pipiens* and *Psorophora columbiae* the presence of *Serratia marcescens*, *Klebsiella ozonae*, *Pseudomonas aeruginosa* and *Enterobacter agglomerans*. Pidiyar *et al*. [18] reported the isolation of a novel *Aeromonas* species from the midgut of *Cx*. *quinquefasciatus*; and Terenius *et al*. [41] found *Aeromonas* sp. in *Anopheles darlingi*. Recently, *Sphingomonas* sp. has been recognized as part of the microbiota of *Culex restuans* and *Cx*. *pipiens*, being more dominant in *Cx*. *restuans* than in *Cx*. *pipiens* adult females [17]. All of these bacteria are commonly isolated from mosquito breeding water [42]; hence, the larval stages can ingest and pass it onto the adult gut [20], and both larvae and adults can obtain it by horizontal transfer, just by being in contact with the contaminated environment (water) [16]. The vertical transmission of these bacteria was never tested in mosquitoes. A relative, *Klebsiella oxytoca* has been found in several other insects, including Diptera species [43]. In *Musca domestica* two major roles have been studied, and this can explain the function of the two bacteria associated with *Cx*. *pipiens*. First, *K*. *oxytoca* provided cues for oviposition, attracting female flies to ovipositing sites, and second, it proliferated on the eggshells. The female flies covered the eggs with *K*. *oxytoca*, which grew over time; however, growth inhibited oviposition after 24 h. The authors [43] did not study larval survival up to pupal stages; therefore, it is not clear if in addition to restraining oviposition the bacterium could also impact survival.

Our results showed that the two bacteria regularly carried by laboratory populations of *Cx*. *pipiens*, strains of *Klebsiella* sp. and *Aeromonas* sp., seem to have a significant effect on female oviposition choice, *Cx*. *pipiens* females significantly preferred substrates inoculated with these two bacteria for oviposition, agreeing with several studies that have shown that bacteria may produce oviposition attractants for different mosquito species [44–46]. Native *Klebsiella* sp. and *Aeromonas* sp. strains isolated in this work, failed in the survival experiments, no complete development was achieved. However, these bacteria were able to properly nurture the very early developmental stages until L2 or day 10 of development (S1 Table), showing that their nutritional value relies on the accessibility of these organisms to the very fragile first instars, neonates. The most vulnerable stage in the life cycle of holometabolous insects is the first instar just after hatching. In a recent study on *Ae*. *aegypti* by Coons *et al*. [16] it was demonstrated that development could only be rescued from sterilized 1^st^ instar larva by inoculating the larvae with native strains only if sterile diet is added as food source. Interestingly, the fish-food (TetraMin) used in Coons *et al*. [16] experiments contains brewer yeast.

Females choose to lay their eggs in habitats that offer the highest probability for their offspring to reach adulthood [47]. For the laboratory colony of *Cx*. *pipiens* it became clear that the instantaneous provision of food, such as *Klebsiella* sp. or *Aeromonas* sp. carried by the mothers at the time of oviposition or being provided by the habitat is what speeds up molting into a more resistant instar. A second larval stage (L2) will be able to find its own food resources, swim faster and will be able to avoid predators. In rich breeding waters containing fungi or yeast, the development of *Cx*. *pipiens* up to adulthood can be secured.

The female mosquitoes selected the two cyanobacteria species used, but the oviposition percentages were low; however, as explained above, cyanobacteria cannot be digested. It has been reported that some cyanobacteria can attract some mosquitoes such as *Anopheles albimanus*, possibly due to an unidentified C-15 aliphatic alcohol found over cyanobacterial mats [48] and for other volatile substances detected by *An*. *albimanus* and *Anopheles vestitipennis* [49]. In experiments using n-pentadecanol, a commercially available compound close in molecular weight and mass spectral pattern to the unknown C-15 aliphatic alcohol, indicated a tendency of *An*. *albimanus* female to oviposit more eggs in containers with the tested chemical when is compared to water [49]. Our results indicated that *Synechosystis* 6803 and *Anabaena* 7120 were inefficient food sources for the mosquito colony and were also not able to transmit to offspring; however, it might have some effect on the oviposition selection. This is, in concordance with Thiery *et al*., [50] who fed *Cx*. *pipiens* and *An*. *gambiae* larvae with some native cyanobacteria strains, including *Synechococcus* PCC 7942, and evaluated their ingestion and digestion, showing that the numbers of cells ingested and digested depended on the cyanobacterial strain and varied with the mosquito species.

In this work we show that *Cx*. *pipiens* can efficiently use *S*. *cerevisiae* as a food source allowing full completeness of development, but not transmission to F1 eggs. This mosquito colony is naturally infected with two different bacteria, *Klebsiella* sp. and *Aeromonas* sp., which are additionally chosen by mosquito females for selection of oviposition sites. Furthermore, these bacteria assure development by providing the very first food intake of neonates.

Control strategies of important widespread vectors such as mosquitoes in the Pipiens Assemblage should be revisited and future methods of mosquito control might take advantage of impact of mosquito native microbiota. Based on these results, we propose the use of a combined strategy by designing baits or attractants based on native-mosquito bacteria (or their volatiles) to attract ovipositing females to artificial yeast-depleted breeding habitats. We continue investigating the mosquito-bacteria interaction, especially transmission patterns as well as the role that these native strains might have in the development of *Cx*. *pipiens*.

## Supporting Information

S1 Fig Developmental time of the immature stages of *Cx*. *pipiens* fed with different microorganisms. Egg rafts from *Cx*. *pipiens* laboratory breeding were washed with non-ionic detergent and then with sterile distilled water for three times. After this, the eggs were placed in plastic containers with sterile distilled water until the emergence of neonate larvae. For each treatment, 20 larvae were transferred to plastic containers with 100 mL of sterile distilled water. Each group was fed with different microorganisms: yeast (labelled GFP-*S*. *cerevisiae*), microalgae (*C*. *sorokiniana*), cyanobacteria (*Anabaena* 7120 and *Synechocystis* 6803), bacteria (*Klebsiella* sp. and *Aeromonas* sp.), and fish food (Shulet ® Carassius) as a control diet. 1 mg of each lyophilized cell cultures was used to feed each group of larvae. The larval development was observed at 5, 10, 15, 20, 23 and 25 days after feeding with each microorganism. The developmental status was determined based on the developmental state of more than the 50% of individuals of each treatment. (A), (B) and (C) correspond to replications 1, 2 and 3 respectively.
(TIF)

S2 Fig Cells of GFP-labelled *S*. *cerevisiae*. Cells were located along the midgut and in gastric ceca of *Cx*. *pipiens* larvae after 24 h of ingestion. Yeast-fed larvae observed by light microscopy (A) and fluorescence microscopy (B). Digestive tracts were removed and observed by light (C) and fluorescence microscopy (D). (M) Midgut and (GC) gastric caeca are indicated by arrows. Bars 100 μm.
(TIF)

S3 Fig Detection of the GFP gene by PCR amplification in different stages of *Cx*. *pipiens*. Insects from the mosquito colony fed on GFP-labelled yeast were washed using sodium hypochlorite, then in sterile distilled water twice, and used for the DNA extraction in order to detect GFP gene by PCR on different stages of *Cx*. *pipiens* (larvae 1 to 4, pupae, adult and eggs). PCR products were electrophoresed on 1% agarose gels. Lanes 1, 2, 3, 4, 5, 6, and 7 correspond to larvae 1 to 4, pupae, female and male adults respectively (M, molecular weight marker, 100 bp ladder, Fermentas®).
(TIF)

S4 Fig Microorganisms isolated from the homogenates of different developmental stages of *Cx*. *pipiens* fed only with GFP-labelled *S*. *cerevisiae*. *S*. *cerevisiae* colony (A) and individual cells observed by light microscopy (B) and by fluorescence microscopy (C). *Klebsiella* sp. colony (D) and individual cells observed by light microscopy (E) and by fluorescence microscopy (F). *Aeromonas* sp. colony (G) and individual cells observed by light microscopy (H) and by fluorescence microscopy (I).
(TIF)

S1 Table Comparison of survival rates for each treatment at day 10 (mosquito development from L2 to L3) and at day 25 (mosquito development from pupae to adult). (DOC)

S1 Video *Cx*. *pipiens* L2 larvae feeding on *Anabaena* 7120. (ZIP)

## References

[1] Becker N, Petrić D, Zgomba M, Boase C, Dahl C, Lane J, et al Mosquitoes and their control. 1st ed. New York, Boston, Dordrecht, London, Moscow: Kulmer Academic/ Plenum Press; 2003.

[2] Laird M. The natural history of larval mosquito habitats London: Academic Press; 1988.

[3] Clements A. The Biology of mosquitoes Development, nutrition, and reproduction. 1st ed. New York: Great Britain at the University Press; 1992.

[4] Douglas AE. The Molecular basis of bacterial-insect symbiosis. J Mol Biol. 2014; 426: 3830–3837. 10.1016/j.jmb.2014.04.005 24735869 PMC4385585

[5] Buchner P. Endosymbiosis of animals with plant microorganims. New York: Interscience Press; 1965.

[6] Vega FE, Dowd PF. The role of yeasts as insect endosymbionts. In: Blackwell M, Vega FE editors. Insect-Fungal Associations: Ecology and Evolution; 2005. pp. 211–243.

[7] Gibson CM, Hunter MS. Extraordinarily widespread and fantastically complex: comparative biology of endosymbiotic bacterial and fungal mutualists of insects. Ecol Lett. 2010; 13: 223–234. 10.1111/j.1461-0248.2009.01416.x 20015249

[8] Ignatova EA, Nagornaia SS, Povazhnaia TN, Ianishevskaia GS. The yeast flora of blood-sucking mosquitoes. Mikrobiol Z. 1996; 58: 12–15. 8983519

[9] Gusmão DS, Santos AV, Marini DC, Russo ÉDS, Peixoto AMD, Bacci M Júnior, et al First isolation of microorganisms from the gut diverticulum of *Aedes aegypti* (Diptera: Culicidae): new perspectives for an insect-bacteria association. Mem Inst Oswaldo Cruz. 2007; 102: 919–924. 18209929 10.1590/s0074-02762007000800005

[10] Ricci I, Mosca M, Valzano M, Damiani C, Scuppa P, Rossi P, et al Different mosquito species host *Wickerhamomyces anomalus* (*Pichia anomala*): perspectives on vector-borne diseases symbiotic control. Anton Leeuw Int J G. 2011; 99: 43–50. 10.1007/s10482-010-9532-3 21113816

[11] Ricci I, Damiani C, Scuppa P, Mosca M, Crotti E, Rossi P, et al The yeast *Wickerhamomyces anomalus* (*Pichia anomala*) inhabits the midgut and reproductive system of the Asian malaria vector *Anopheles stephensi*. Environ Microbiol. 2011; 13: 911–921. 10.1111/j.1462-2920.2010.02395.x 21208355

[12] Gusmão DS, Santos AV, Marini DC, Bacci M, Berbert-Molina MA, Lemos FJA. Culture-dependent and culture-independent characterization of microorganisms associated with *Aedes aegypti* (Diptera: Culicidae) (L.) and dynamics of bacterial colonization in the midgut. Acta Trop. 2010; 115: 275–281. 10.1016/j.actatropica.2010.04.011 20434424

[13] Ricci I, Damiani C, Capone A, DeFreece C, Rossi P, Favia G. Mosquito/microbiota interactions: from complex relationships to biotechnological perspectives. Curr Opin Microbiol. 2012; 15: 278–284. 10.1016/j.mib.2012.03.004 22465193

[14] Duguma D, Rugman-Jones P, Kaufman MG, Hall MW, Neufeld JD, Stouthamer R, et al Bacterial communities associated with *Culex mosquito* larvae and two emergent aquatic plants of bioremediation importance. PLoS One. 2013; 8: e72522 10.1371/journal.pone.0072522 23967314 PMC3744470

[15] Duguma D, Hall MW, Rugman-Jones P, Stouthamer R, Terenius O, Neufeld JD. Developmental succession of the microbiome of *Culex* mosquitoes. BMC Microbiol. 2015; 15: 1. 26205080 10.1186/s12866-015-0475-8 PMC4513620

[16] Coon KL, Vogel KJ, Brown MR, Strand MR. Mosquitoes rely on their gut microbiota for development. Mol Ecol. 2014; 23: 2727–2739. 10.1111/mec.12771 24766707 PMC4083365

[17] Muturi EJ, Kim CH, Bara J, Bach EM, Siddappaji MH. *Culex pipiens* and *Culex restuans* mosquitoes harbor distinct microbiota dominated by few bacterial taxa. Parasit Vectors. 2016; 9: 1–11. 26762514 10.1186/s13071-016-1299-6 PMC4712599

[18] Pidiyar VJ, Jangid K, Patole MS, Shouche YS. Studies on cultured and uncultured microbiota of wild *Culex quinquefasciatus* mosquito midgut based on 16S ribosomal RNA gene analysis. Am J Trop Med Hyg. 2004; 70: 597–603. 15210998

[19] Wang Y, Gilbreath TM, Kukutla P, Yan G, Xu J. Dynamic gut microbiome across life history of the malaria mosquito *Anopheles gambiae* in Kenya. PloS One. 2011; 6: e24767 10.1371/journal.pone.0024767 21957459 PMC3177825

[20] Osei-Poku J, Mbogo CM, Palmer WJ, Jiggins FM. Deep sequencing reveals extensive variation in the gut microbiota of wild mosquitoes from Kenya. Mol Ecol. 2012; 21: 5138–5150. 10.1111/j.1365-294X.2012.05759.x 22988916

[21] Chavshin AR, Oshaghi MA, Vatandoost H, Pourmand MR, Raeisi A, Terenius O. Isolation and identification of culturable bacteria from wild *Anopheles culicifacies*, a first step in a paratransgenesis approach. Parasit Vectors. 2014; 1: 419. 10.1186/1756-3305-7-419 PMC4261757 25189316

[22] Vinogradova EB. *Culex pipiens pipiens* mosquitoes: taxonomy, distribution, ecology, physiology, genetics, applied importance and control. Bulgaria: Pensoft Press; 2000.

[23] Farajollahi A, Fonseca DM, Kramer LD, Kilpatrick AM. “Bird biting” mosquitoes and human disease: a review of the role of *Culex pipiens* complex mosquitoes in epidemiology. Infect Genet Evol. 2011; 11: 1577–1585. 10.1016/j.meegid.2011.08.013 21875691 PMC3190018

[24] Rossi GC, Mariluis JC, Schnack JA, Spinelli GR. Dípteros vectores (Culicidae y Calliphoridae) de la provincia de Buenos Aires. La Plata: ProBiota, Cobiobo Press; 2002.

[25] Rossi GC. Annotated checklist, distribution, and taxonomic bibliography of the mosquitoes (Insecta: Diptera: Culicidae) of Argentina. Check List. 2015; 11: 1712.

[26] Flores FS, Diaz LA, Batallán GP, Almirón WR, Contigiani MS. Vertical transmission of St. Louis encephalitis virus in *Culex quinquefasciatus* (Diptera: Culicidae) in Córdoba, Argentina. Vector Borne Zoonotic Dis. 2010; 10: 999–1002. 10.1089/vbz.2009.0136 20426683

[27] Micieli MV, Matacchiero AC, Muttis E, Fonseca DM, Aliota MT, Kramer LD. Vector competence of Argentine mosquitoes (Diptera: Culicidae) for West Nile virus (Flaviviridae: Flavivirus). J Med Entomol. 2013; 50: 853–862. 23926785 10.1603/me12226 PMC3932752

[28] Sambrook J, Russell DW. Molecular cloning. A laboratory manual. 1rd ed. New York: Cold pring Harbor Laboratory Press; 2001.

[29] Rippka R. Isolation and purification of cyanobacteria. Methods Enzymol. 1988; 167: 3 3148836 10.1016/0076-6879(88)67004-2

[30] Nikoh N, Fukatsu T. Interkingdom host jumping underground: phylogenetic analysis of entomoparasitic fungi of the genus *Cordyceps*. Mol Biol Evol. 2000; 17: 629–638. 10742053 10.1093/oxfordjournals.molbev.a026341

[31] Weisburg WG, Barns SM, Pelletier DA, Lane DJ. 16S ribosomal DNA amplification for phylogenetic study. J Bacteriol. 1991; 173: 697–703. 1987160 10.1128/jb.173.2.697-703.1991 PMC207061

[32] Stata Corp. Stata Statistical Software, Release 14. College Station, TX: Stata Corp LP 2015.

[33] IBM Corp. IBM SPSS Statistics for Windows, Version 20.0. Armonk, NY: IBM Corp 2011.

[34] Khan I, Farid A, Zeb A. Development of inexpensive and globally available larval diet for rearing *Anopheles stephensi* (Diptera: Culicidae) mosquitoes. Parasit Vectors. 2013; 6: 90 10.1186/1756-3305-6-90 23570246 PMC3626612

[35] Balestrino F, Puggioli A, Gilles JRL, Bellini R. Validation of a new larval rearing unit for *Aedes albopictus* (Diptera: Culicidae) mass rearing. PloS One. 2014; 9: e91914 10.1371/journal.pone.0091914 24647347 PMC3960149

[36] Chapman R. The Insects: Structure and Function. Cambridge: Cambridge University Press; 1998.

[37] Merritt RW, Dadd RH, Walker ED. Feeding behavior, natural food, and nutritional relationships of larval mosquitoes. Annu Rev Entomol. 1992; 37: 349–374. 1347208 10.1146/annurev.en.37.010192.002025

[38] Howland LJ. The nutrition of mosquito larvae, with special reference to their algal food. Bull Entomol Res. 1930; 21: 431–439.

[39] Marten GG. Larvicidal algae.). J Am Mosq Control Assoc. 2007; 23: 177–183. 10.2987/8756-971X(2007)23[177:LA]2.0.CO;2 17855939

[40] Demaio J, Pumpuni CB, Kent M, Beier JC. The midgut bacterial flora of wild *Aedes triseriatus*, *Culex pipiens*, and *Psorophora columbiae* mosquitoes. Am J Trop Med Hyg. 1996; 54: 219–223. 8619452 10.4269/ajtmh.1996.54.219

[41] Terenius O, De Oliveira CD, Pinheiro WD, Tadei WP, James AA, Marinotti O. 16S rRNA gene sequences from bacteria associated with adult *Anopheles darlingi* (Diptera: Culicidae) mosquitoes. J Med Entomol. 2008; 45: 172–175. 18283961 10.1603/0022-2585(2008)45[172:srgsfb]2.0.co;2

[42] Smith TW, Walker ED, Kaufman MG. Bacterial density and survey of cultivable heterotrophs in the surface water of a freshwater marsh habitat of *Anopheles quadrimaculatus* larvae (Diptera: Culicidae). J Am Mosq Control Assoc. 1998; 14: 72–77. 9599327

[43] Lam K, Babor D, Duthie B, Babor EM, Moore M, Gries G. Proliferating bacterial symbionts on house fly eggs affect oviposition behaviour of adult flies. Anim Behav. 2007; 74: 81–92.

[44] Trexler JD, Apperson CS, Zurek L, Gemeno C, Schal C, Kaufman M, et al Role of bacteria in mediating the oviposition responses of *Aedes albopictus* (Diptera: Culicidae). J Med Entomol. 2003; 6: 841–848. 10.1603/0022-2585-40.6.841 14765661

[45] Sumba LA, Guda TO, Deng AL, Hassanali A, Beier JC, Knols BG. Mediation of oviposition site selection in the African malaria mosquito *Anopheles gambiae* (Diptera: Culicidae) by semiochemicals of microbial origin. Int J Trop Insect Sci. 2004; 24: 260–265.

[46] Lindh JM, Borg-Karlson AK, Faye I. Transstadial and horizontal transfer of bacteria within a colony of *Anopheles gambiae* (Diptera: Culicidae) and oviposition response to bacteria-containing water. Acta trop. 2008; 107: 242–250. 10.1016/j.actatropica.2008.06.008 18671931

[47] Ponnusamy L, Böröczky K, Wesson DM, Schal C, Apperson CS. Bacteria stimulate hatching of yellow fever mosquito eggs. PLoS One. 2011; 6: e24409 10.1371/journal.pone.0024409 21915323 PMC3167859

[48] Rejmankova E, Higashi RM, Roberts DR, Lege M, Andre RG. The use of solid phase microextraction (SPME) devices in analysis for potential mosquito oviposition attractant chemicals from cyanobacterial mats. Aquat Ecol. 2000; 34: 413–420.

[49] Rejmánková E, Higashi R, Grieco J, Achee N, Roberts D. Volatile substances from larval habitats mediate species-specific oviposition in *Anopheles* mosquitoes. J Med Entomol. 2005; 42: 95–103. 15799516 10.1093/jmedent/42.2.95

[50] Thiery I, Nicolas L, Rippka R, de Marsac NT. Selection of cyanobacteria isolated from mosquito breeding sites as a potential food source for mosquito larvae. Appl Environ Microbiol. 1991; 57: 1354–1359. 1677241 10.1128/aem.57.5.1354-1359.1991 PMC182954

