# Supplementary figures and images for: Culex pipiens Development Is Greatly Influenced by Native Bacteria and Exogenous Yeast
Source: PLoS One. 2016 Apr 7;11(4):e0153133. doi: 10.1371/journal.pone.0153133 (PMC4824439; doi:10.1371/journal.pone.0153133)

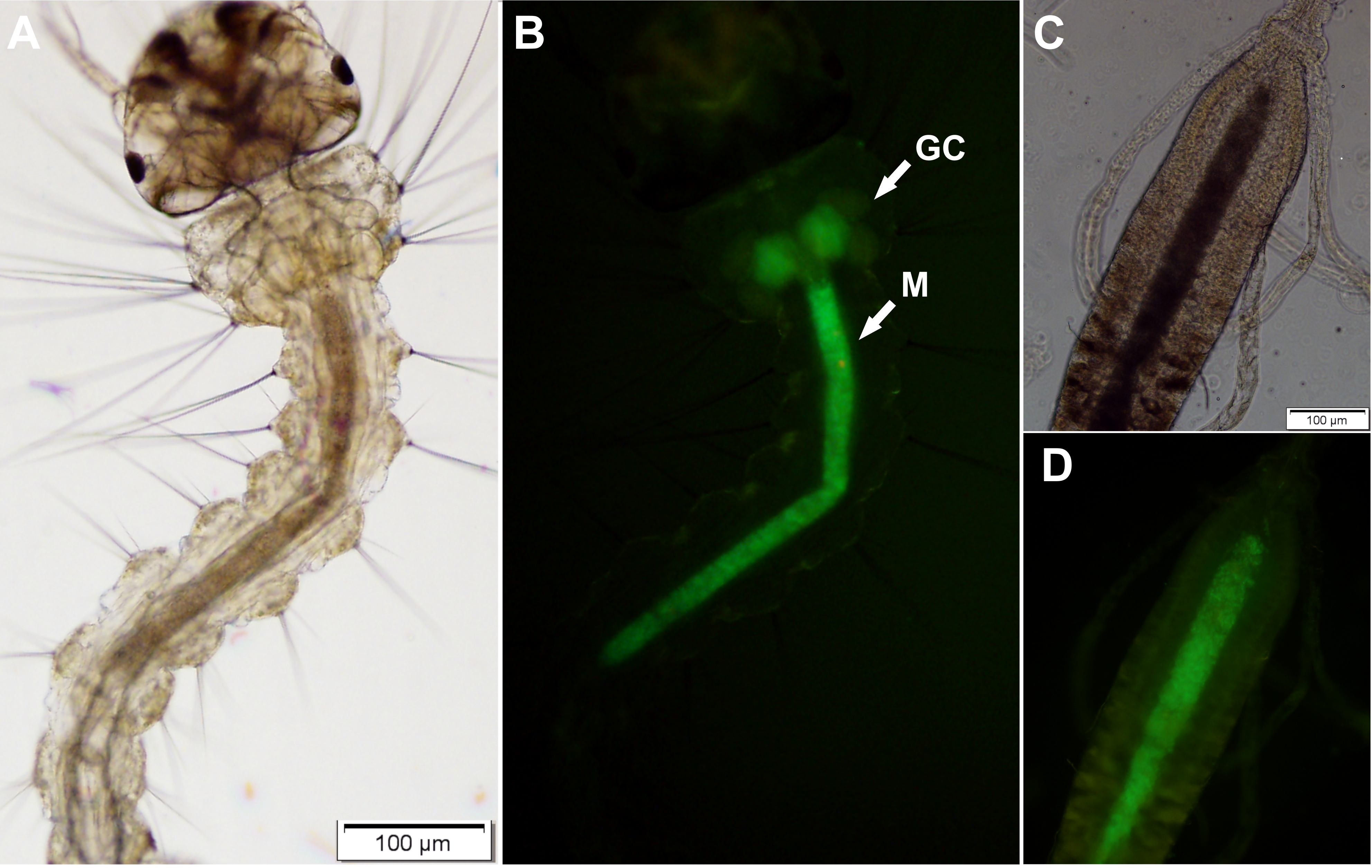

Supplement: S2 Fig — Cells were located along the midgut and in gastric ceca of Cx. pipiens larvae after 24 h of ingestion. Yeast-fed larvae observed by light microscopy (A) and fluorescence microscopy (B). Digestive tracts were removed and observed by light (C) and fluorescence microscopy (D). (M) Midgut and (GC) gastric caeca are indicated by arrows. Bars 100 μm. (TIF) [file pone.0153133.s002.tif]

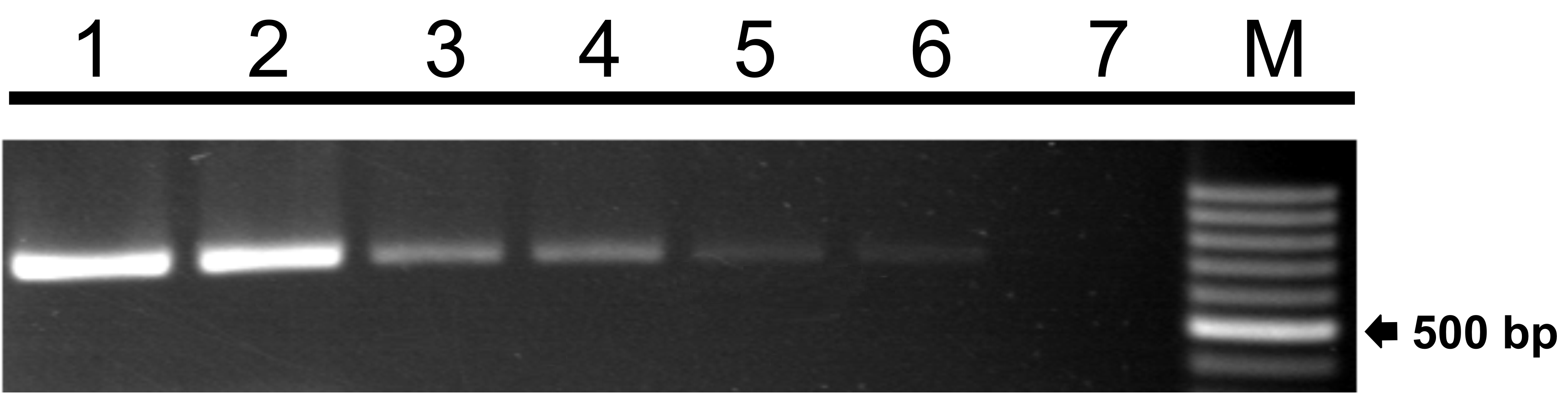

Supplement: S3 Fig — Insects from the mosquito colony fed on GFP-labelled yeast were washed using sodium hypochlorite, then in sterile distilled water twice, and used for the DNA extraction in order to detect GFP gene by PCR on different stages of Cx. pipiens (larvae 1 to 4, pupae, adult and eggs). PCR products were electrophoresed on 1% agarose gels. Lanes 1, 2, 3, 4, 5, 6, and 7 correspond to larvae 1 to 4, pupae, female and male adults respectively (M, molecular weight marker, 100 bp ladder, Fermentas®). (TIF) [file pone.0153133.s003.tif]

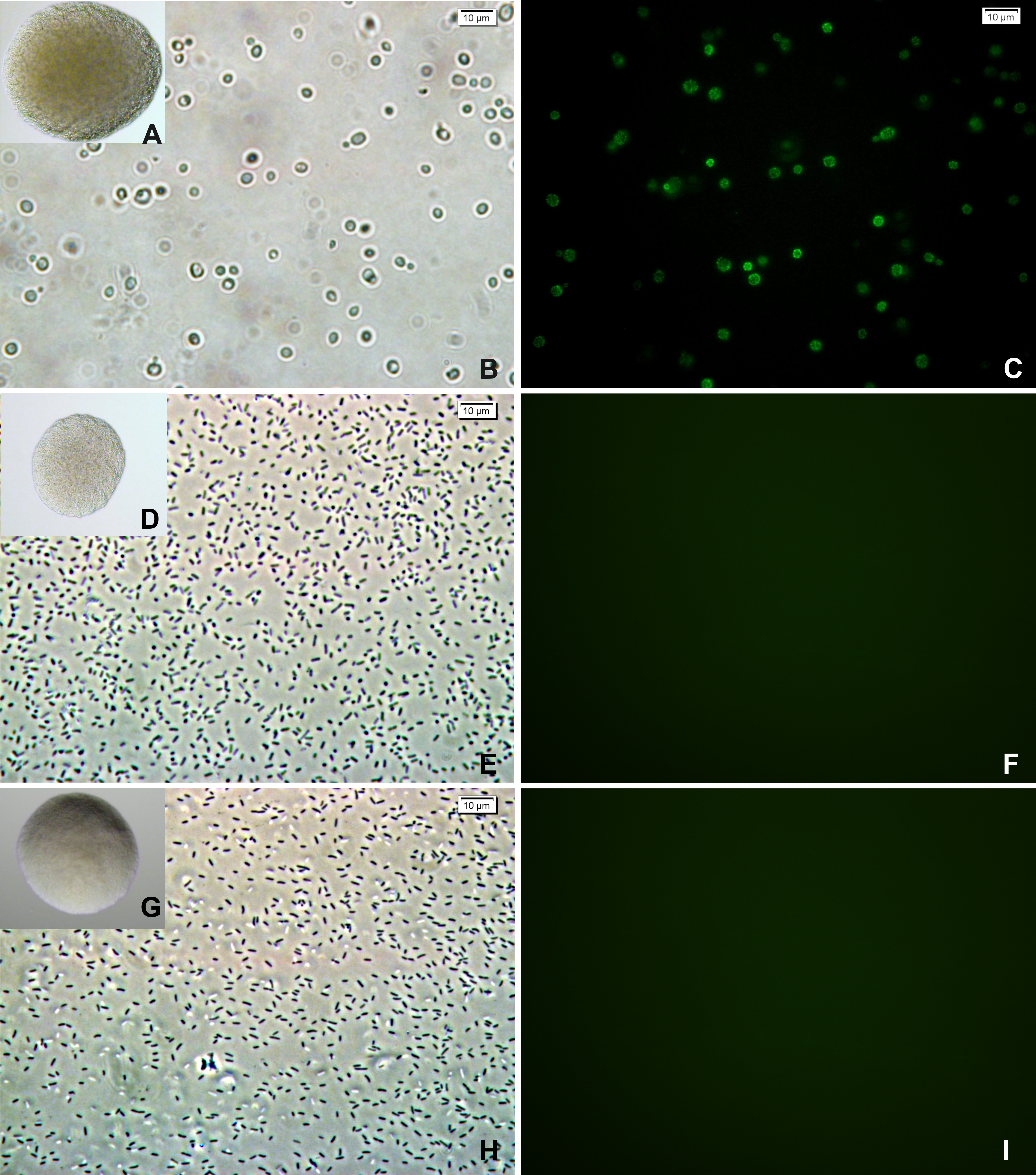

Supplement: S4 Fig — S. cerevisiae colony (A) and individual cells observed by light microscopy (B) and by fluorescence microscopy (C). Klebsiella sp. colony (D) and individual cells observed by light microscopy (E) and by fluorescence microscopy (F). Aeromonas sp. colony (G) and individual cells observed by light microscopy (H) and by fluorescence microscopy (I). (TIF) [file pone.0153133.s004.tif]
