# Supplementary material for: Culex pipiens Development Is Greatly Influenced by Native Bacteria and Exogenous Yeast
Source: PLoS One. 2016 Apr 7;11(4):e0153133. doi: 10.1371/journal.pone.0153133 (PMC4824439; doi:10.1371/journal.pone.0153133)
Supplement: S1 Table — (DOC) [file pone.0153133.s005.doc]

|  | **Survival Rate**  **at Day 10** | | | **Survival Rate**  **at Day 25** | |
| --- | --- | --- | --- | --- | --- |
| **Treatments** | **N** | **Mean ± SD (*)** | **N** | | **Mean ± SD (*)** |
| Control | 160 | 0.94 ± 0.05 a | 160 | | 0.87 ± 0.16 a |
| *S. cerevisiae* | 160 | 0.94 ± 0.07 a | 160 | | 0.87 ± 0.14 a |
| *C. sorokiniana* | 160 | 0.53 ± 0.37 b | 160 | | 0 |
| *Anabaena* 7120 | 160 | 0.32 ± 0.46 b | 160 | | 0 |
| *Sinechocystis* 6803 | 160 | 0.31 ± 0.46 b | 160 | | 0 |
| *Klebsiella* sp. | 160 | 0.32 ± 0.45 b | 160 | | 0 |
| *Aeromonas* sp. | 160 | 0.30 ± 0.43 b | 160 | | 0 |

**S1 Table.** Comparison of survival rates for each treatment at day 10 (mosquito development from L2 to L3) and at day 25 (mosquito development from pupae to adult).

(*) Means followed by different letters are significantly different at P = 0.05
